# Reappraisal of clinical data supports double IUI for improved pregnancy outcomes

**Published:** 2018-03

**Authors:** G Bahadur, R Homburg

**Affiliations:** Reproductive Medicine Unit, North Middlesex University Hospital, Old Admin Block, Sterling Way, London N18 1QX, UK; Homerton Fertility Unit, Homerton University Hospital, Homerton Row, London E9 6SR, UK

**Keywords:** double IUI, infertility, male factor, pregnancy success

## Abstract

Optimising pregnancy and live birth outcomes for fertility procedures is highly desirable and involves disentangling numerous potentially contributing factors. In IUI procedures would double inseminations within a cycle be beneficial? Despite mistaken belief amongst the fertility practitioners the available evidence including Cochrane review has suggested, there would be beneficial effects of utilising double IUI within a cycle. Here we examine new evidence attempting to clarify the role of double versus single IUI.

IUI is less stressful, less invasive and less expensive than IVF in our opinion and evidence from some patient populations supports IUI as a first line treatment option over IVF (Bahadur et al., 2016; Tjon-Kon-Fat et al., 2015; Ombelet et al., 2017; Farquhar et al., 2018). Improving IUI outcomes has been disproportionately overlooked compared with more expensive procedures (Heneghan et al., 2016). The value of IUI as first line treatment and ways of optimising outcomes is reported (Bahadur et al., 2017a).

If sperm is available at the right time for insemination then fertilisation is likely to occur and the idea of multiple inseminations during a cycle could help capture the right moment for fertilisation and increase the pregnancy rates. Would doing two IUI’s on successive days (double IUI) over single IUI therefore be beneficial? Practitioners have long dismissed the benefits of multiple inseminations within a cycle based on a Cochrane review involving three studies and 386 women, which showed no benefit of double IUI (OR 1.45, 95% CI 0.78-2.70) (Cantineau, 2003a). The updated intervention review in October 2007 incorporated six studies and 1785 women (Cantineau, 2003b). One study was excluded as the data was replicated. The results of five studies that reported pregnancy rate per couple showed a significant effect of using double insemination (Peto odds ratio 1.8, 95% confidence interval 1.4 to 2.4). In conclusion, double intrauterine insemination resulted in significant benefit over single intrauterine insemination in the treatment of subfertile couples with husband semen. The validity of a meta-analysis is highly dependent on the quality and inclusion/exclusion criteria, the subjective level of quality assigned to studies and grading being biased toward RCT and the choice of statistical analyses (Alikani et al., 2017). A parallel analysis wrongly promoted IVF over IUI through the NICE guidelines in the absence of evidence (Bahadur et al., 2017b).

Numerous studies post Cochrane review shows the beneficial effects of double IUI over single IUI. In one study, there was a two-fold significant increase in pregnancies after a cycle with a double IUI compared to single IUI (OR: 2.0; 95% CI: 1.07-3.75; P<0.03) but further large and well-designed randomized studies were requested (Zavos et al., 2013). In another report with 865 patients and 1156 cycles, the pregnancy rate/cycle in the two-insemination/-cycle group (14.9% vs. 11.4%), was without statistically significant differences (RR = 1.34; 95% confidence interval 0.90-1.99) (Osuna et al., 2004).

Interestingly, gonadotropin (Gn) stimulated cycles, ovulatory dysfunction and male factor diagnostic categories were favourable for double IUI; between single and double IUI groups (ovulation dysfunction, 12.9% vs 19.5%, p < 0.048, and male factor, 7.9% vs. 17.5%, p < 0.030) and ovulation protocols (Clomiphene citrate (CC)-Gn-human chorionic gonadotropin (hCG), 13.0% vs. 21.3%, p < 0.031, and L-Gn-hCG, 4.2% vs. 25.0%, p < 0.002)( Randall and Gantt, 2008). Male factor patients had significant benefit receiving double IUI over single IUI, 19.8% and 11.06% (p < 0.05), respectively, whereas there was no significant difference within idiopathic infertility groups (10.5% vs. 11.9%, p > 0.05) (Liu et al., 2006). Double IUI compared with single IUI gave a better PR and the OR for all cycles was 1.9 (0.76-4.7) (P = 0.22), but according to etiology, it was 4.7 (0.9-24.13) (P = 0.06) in male factor and 1.2 (0.43- 3.33) (P = 0.779) for non-male factors (Ghanem et al., 2011).

In the unexplained infertility group the LBR was 11.1% (5/45 patients) with single IUI and 18.4% (9/49) with double IUI (P = 0.393) (Bagis et al., 2010). Costings issues will prevail but this must be placed in the context of how successfully pregnancy rates are achieved within each clinic. This is nicely demonstrated in that cost effectiveness can be gained with the use of the more expensive gonadotrophins over CC if more pregnancies can be achieved early (Peeraer et al., 2017). No cost analysis of single versus double IUI has been performed yet.

In conclusion, the available evidence including the Cochrane review did support the use of a double IUI practice within a cycle. Newer data further suggests that male factor infertility and gonadotrophin induced cycles may benefit from double IUI and more systematic studies would be desirable.
